# Supplementary material for: Predicting visual function by interpreting a neuronal wiring diagram
Source: Nature. 2024 Oct 2;634(8032):113–23. doi: 10.1038/s41586-024-07953-5 (PMC11446822; doi:10.1038/s41586-024-07953-5)
Supplement: Supplementary file 6 — CRF and ERF predictions for individual TmY4 and TmY9 cells. Analogous to Supplementary Data 3, but for TmY target types. Shown are the top four monosynaptic pathways, the strongest pathway passing through each of the top ten intermediary types (ranking from Extended Data Fig. 7), and the trisynaptic pathway Tm1–TmY–Dm3–TmY (see the section entitled Prediction of spatial normalization). [file 41586_2024_7953_MOESM6_ESM.zip › DataS4/TmY9q/720575940624161207.pdf]

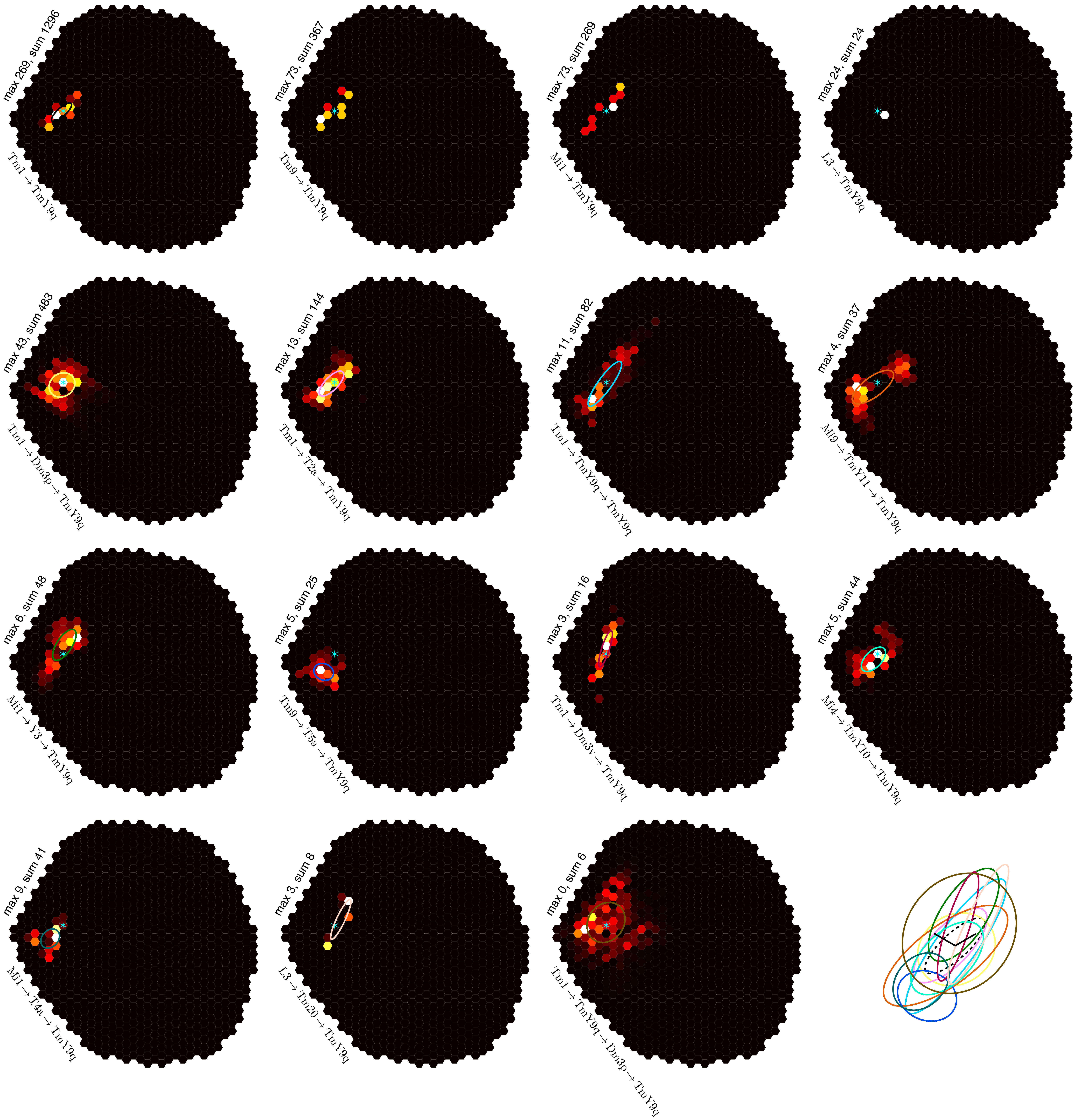
max 269, sum 1296
Tm1 → TmY9q
max 73, sum 367
Tm9 → TmY9q
max 73, sum 269
Mi1 → TmY9q
max 24, sum 24
L3 → TmY9q
max 43, sum 483
Tm1 → Dm3p → TmY9q
max 13, sum 144
Tm1 → T2a → TmY9q
max 11, sum 82
Tm1 → TmY9q → TmY9q
max 4, sum 37
Mi9 → TmY11 → TmY9q
max 6, sum 48
Mi1 → Y3 → TmY9q
max 5, sum 25
Tm9 → T5a → TmY9q
max 3, sum 16
Tm1 → Dm3v → TmY9q
max 5, sum 44
Mi4 → TmY10 → TmY9q
max 9, sum 41
Mi1 → T4a → TmY9q
max 3, sum 8
L3 → Tm20 → TmY9q
max 0, sum 6
Tm1 → TmY9q → Dm3p → TmY9q
